# Intergenerational Relationships as a Resource for Resilience During the COVID-19 Pandemic

**DOI:** 10.1093/geroni/igab046.504

**Published:** 2021-12-17

**Authors:** Shelbie Turner, Karen Hooker, Shannon Jarrott, John Geldhof

**Affiliations:** 1 Oregon State University, Oregon State University, Oregon, United States; 2 Oregon State University, Corvallis, Oregon, United States; 3 The Ohio State University, Columbus, Ohio, United States

## Abstract

The intergenerational ties that offer support to older adults are likely useful for resilience during the COVID-19 pandemic. We analyzed whether positive and negative intergenerational contact was associated with positive pandemic-related personal change. We utilized data collected from 566 adults aged 50 and older between August 2020 and January 2021 via MTurk and a statewide research registry. Participants reported the quality of their contact with younger adults, and whether they experienced positive changes (i.e. new hobbies, healthier behavior, greater meaning in work) as a result of the pandemic. Higher positive, but not lower negative, non-familial intergenerational contact was associated with a higher number of positive pandemic-related changes (estimate = 0.07, SE = 0.03, p=0.02). The quality of familial intergenerational relationships were not associated with positive pandemic-related changes. Non-familial intergenerational relationships may be especially important for resilience, and should be supported during a time when they may be difficult to maintain.

